# CD8 T Cell–Independent Antitumor Response and Its Potential for Treatment of Malignant Gliomas

**DOI:** 10.3390/cancers8080071

**Published:** 2016-07-27

**Authors:** Katherine A. Murphy, Thomas S. Griffith

**Affiliations:** 1 Department of Urology, University of Minnesota, Minneapolis, MN 55455, USA; tgriffit@umn.edu; 2 Center for Immunology, University of Minnesota, Minneapolis, MN 55455, USA; 3 Masonic Cancer Center, University of Minnesota, Minneapolis, MN 55455, USA

**Keywords:** brain tumors, immunotherapy, T cells

## Abstract

Malignant brain tumors continue to represent a devastating diagnosis with no real chance for cure. Despite an increasing list of potential salvage therapies, standard-of-care for these patients has not changed in over a decade. Immunotherapy has been seen as an exciting option, with the potential to offer specific and long lasting tumor clearance. The “gold standard” in immunotherapy has been the development of a tumor-specific CD8 T cell response to potentiate tumor clearance and immunological memory. While many advances have been made in the field of immunotherapy, few therapies have seen true success. Many of the same principles used to develop immunotherapy in tumors of the peripheral organs have been applied to brain tumor immunotherapy. The immune-specialized nature of the brain should call into question whether this approach is appropriate. Recent results from our own experiments require a rethinking of current dogma. Perhaps a CD8 T cell response is not sufficient for an organ as immunologically unique as the brain. Examination of previously elucidated principles of the brain’s immune-specialized status and known immunological preferences should generate discussion and experimentation to address the failure of current therapies.

## 1. Introduction

Despite the growing interest in immunotherapy for brain tumors, the prognosis for this disease remains grim. There are approximately 22,000 malignant primary brain tumors diagnosed each year in the United States, with malignant gliomas accounting for the majority of these cases [1]. Although brain tumors do not account for the majority of cancer diagnoses, they do represent a disproportionately high number of cancer deaths [2,3]. Patients with grade IV gliomas, referred to as glioblastoma multiforme (GBM) have a median survival of 15–19 months [1]. This dismal prognosis is due in part to the lack of therapeutic options for patients diagnosed with these tumors. The current standard-of-care for GBM includes surgical resection, radiotherapy, and chemotherapy. The unique nature of the brain has posed a challenge to the development of additional therapeutic options. The blood brain barrier (BBB) can exclude certain drugs, making some of the pharmaceutics developed for other tumors unusable. Additionally, due to the invasive nature of the tumor, surgical resection of the entire tumor has proven to be impossible, as tumor cells are able to infiltrate into normal brain tissues [4]. Even in extreme cases where full hemispherectomies were performed the tumor ultimately recurred in the contralateral hemisphere [5]. The current chemotherapy of choice is temozolomide (TMZ), primarily due to its ability to penetrate the BBB [6]. The combination of TMZ and radiotherapy is more effective at extending survival than radiotherapy alone, resulting in progression-free survival of 11% at two years compared to 1.8% for radiotherapy alone. However, 5-year progression-free survival for the TMZ/radiotherapy combination is still a dismal 4.1% [7]. Even when therapy is successful at extending survival, the side effects can be devastating. All the current methods used can result in damage to the surrounding normal tissues and cause long-term neurological problems. This is especially problematic in pediatric patients whose developing nervous system is particularly susceptible to this bystander effect [8,9].

Immunotherapy is an attractive option for brain tumors, offering the potential for specific and enduring tumor clearance. The complex nature of the immune response in the central nervous system (CNS) poses a unique challenge to the field of brain tumor immunotherapy. The generation of a tumor-specific CD8 T cell response has been a focus of tumor immunotherapy. While this approach is well reasoned the results have been disappointing, producing only minimal responses in patients and no evidence of cure. One possible explanation for the limited success with current immunotherapy protocols is the failure to target other components of the immune system. Examination of the immunological status of the CNS and the potential to elicit alternative immune responses against tumors may provide an explanation for suboptimal responses and offer another means for tumor elimination.

## 2. Immunology in the CNS

Brain tumor immunotherapy is especially challenging due to the specialized relationship between the CNS and the immune system. The idea of an immune privileged state in the CNS developed from early studies that analyzed differential rejection of transplanted tissue in the CNS versus periphery. Tumors that were readily rejected in peripheral organs were protected from immunological rejection in the brain parenchyma suggesting a lack of immunological response [10]. The apparent (recently refuted [11]) lack of lymphatic vessels in the brain and the presence of the BBB supported the notion of an immunologically sterile site. Additional studies demonstrated transplanted tissues could be readily rejected in the CNS if the host had previously rejected the graft in the periphery; suggesting activated lymphocytes were able to migrate into the CNS. However, this data suggested a paucity of lymphatic drainage from the CNS prevented the rejection of transplants in non-immunized hosts [12]. Further work demonstrated grafts implanted into the brain resulted in accelerated immune-rejection of these grafts in the periphery, suggesting the CNS was indeed capable of eliciting an immune response outside of the CNS [13]. These data collectively suggest the afferent and efferent arms of the immune system are intact in the CNS, contrary to initial hypotheses. More recent studies have examined the efflux of proteins out of the CNS and found fluid (carrying potential antigens) leaving the brain enters the blood or drains to the ipsilateral cervical lymph nodes, with a preference to the cervical lymph nodes [14]. The preferential drainage of the CNS to the cervical lymph nodes makes this an important site for brain tumor immunology [14]. Vaccination near this site is appealing as priming at the cervical lymph nodes could boost endogenous antigen presentation and take advantage of the existing proximity and bias to the brain. Prior studies have detailed the cervical lymph nodes and CNS response to antigen in great detail [14]. While the previous theory of an “immune privileged” status has been disputed a new concept in neuroimmunology has emerged. The “immune-specialized” nature of the CNS takes into account the many differences of the immune response in the CNS compared with peripheral organs. Cervical lymph nodes are predisposed to induce a Th2 immune response, preferentially activating a humoral immune response at the expense of a cytotoxic T lymphocyte (CTL) response [15]. Although CD8 T cells in the cervical lymph nodes do respond to antigen and undergo rapid proliferation, direct CTL activity was not observed [16]. CTL suppression in the brain is likely due to CNS production of transforming growth factor (TGF)-β, which suppresses CTL activity rendering this response ineffective [17]. Additionally, cerebral spinal fluid (CSF) draining to the cervical lymph nodes contains TGF-β possibly generating the polarized response. Moreover, in viral infections of immune specialized sites, such as the lung or brain, activated CD8 T self-regulate by producing the anti-inflammatory cytokine IL-10 potentially to minimize tissue destruction [18]. The physical nature of the brain does not permit much swelling or inflammation without catastrophic consequences, and it is reasonable to assume the tight regulation of the inflammatory immune response serves to limit damage to such a significant organ. Considering these restraints, immunotherapy for tumors within the CNS requires overcoming significant hurdles.

## 3. Immunotherapy in Cancer

A variety of approaches designed to stimulate a tumor-reactive immune response have been examined in a multitude of tumor types. Despite the complex nature of CNS immunology, immunotherapy for the treatment of brain tumors is still an active area of research. The FDA approval of the cancer immunotherapies Provenge^®^ (sipuleucel-T, for prostate cancer) and ipilimumab (for metastatic melanoma) have provided an initial proof-of concept for effective antitumor immune response [19,20]. More recently, therapies targeting PD-1 and PD-L1 have demonstrated exciting results; FDA approval for nivolumab and pembrolizumab (anti-PD-1) has further bolstered the concept of immunotherapy being a viable treatment option for cancer patients [21]. However, the shortage of successful immunotherapies at large underscores the difficulties the field as a whole has faced. This is further emphasized by the paucity of phase III clinical trials for brain tumor immunotherapy.

### 3.1. Monoclonal Antibodies (mAb)

Delivery of mAb have been used to target known tumor associated antigens. Human epidermal growth factor 2 (Her2) is overexpressed in approximately 30% of breast cancers and administration of Herceptin^®^ (trastuzumab, a monoclonal antibody against Her2) has shown efficacy in Her2 positive breast cancer patients [22]. Likewise, epidermal growth factor receptor variant III (EGFRvIII), a common mutation associated with glioma, results in a novel peptide sequence that serves as a neoantigen present in approximately 30% of GBM patients [23]. Treatment of mouse models with antibodies targeted to EGFRvIII have resulted in tumor regression and similar therapies have entered clinical trials [24]. Passive immunotherapy approaches lack both breadth of coverage and immunological memory. Without defined neoantigens for every individual tumor this therapy fails to be accessible for many patients. Additionally, heterogeneity within the tumors themselves allows for immune evasion in the absence of multiple targets.

### 3.2. Adoptive Cell Transfer (ACT)

ACT has been most successful in the field of melanoma immunotherapy, where tumor infiltrating lymphocytes (TIL) are expanded ex vivo and re-administered to patients upon sufficient proliferation [25]. ACT for brain tumors using lymphocytes isolated from peripheral blood and tumor has been attempted in several clinical trials with disappointing results [26]. The focus on ACT has progressed to testing genetically engineered autologous T cells to express chimeric antigen receptors (CAR) designed to target specific tumor antigens [27]. Targeting CD19 in clinical trials with acute lymphoblastic leukemia (ALL) showed remarkable success [28]. Building on that success researchers have engineered CAR T cells specific for HER2, EPHA 2, IL13RA2, and EGFRvIII [29,30,31,32,33]. Success in mouse models has led to a human clinical trials for EGFRvIII and IL13RA2 specific CAR therapy [34,35]. Similar to mAb, this type of therapy only benefits those individuals whose tumors express the specific proteins in which the CAR T cells are specific for. Identification of additional antigenic targets will allow application to a greater pool of patients.

### 3.3. Immune Modulators

Ipilimumab (Yervoy^®^) is a humanized mAb against cytotoxic T lymphocyte antigen 4 (CTLA-4), a regulatory receptor upregulated on activated T cells, which received FDA approval for the treatment of metastatic melanoma. CTLA-4 competes with CD28 for CD80/86 ligation and effectively blocks IL-2 production and proliferation [36]. By preventing CTLA-4 from binding to CD80/86, ipilimumab allows for sustained signaling through CD28 to occur without inhibition, effectively “removing the brakes” from the immune response. In clinical trials many patients saw increased progression-free survival and rarely complete response; however, the majority of patients also development severe autoimmune complications [37].

FDA approval of ipilimumab restimulated the field of immunotherapy and additional checkpoint inhibitors are being investigated for potential clinical use in brain tumors. Like CTLA-4, the programed death (PD) receptor pathway has emerged as a potential target for immunotherapy. PD-1 is expressed on activated T cells and binding to one of its ligands (PD-L1 or PD-L2) results in decreased T cell activity [38,39]. PD-L1 expression on tumors is believed to promote immune evasion, and expression has been noted on several types of tumors, including glioma [40]. Several mouse glioma models have demonstrated efficacy using mAb against either PD-1 or PD-L1 and both of these therapies have entered clinical trials for brain tumors [41,42,43,44].

The use of immune modulatory agents for cancer treatment has been rapidly incorporated into pre-clinical immunotherapy models. Blocking CLTA-4 or PD-1/PD-L1 allows for the potentiation of an antitumor response in a non-specific manner. Additionally, there are many more immune modulators that are promising targets; following TCR-MHC engagement and CD28 ligation by CD80/86 several additional costimulatory molecules are upregulated on the surface of the APCs and T cells. These additional costimulatory molecules include members of the tumor necrosis factor receptor (TNFR) family, such as 4-1BB, glucocorticoid-induced TNFR (GITR), and OX40. Several approaches have been used to activate costimulatory signaling pathways including, agonistic mAb and ligand fusion proteins, where the corresponding ligands are fused to immunoglobulin proteins for systemic delivery in vivo [45]. Manipulation of these costimulatory molecules has been attempted in animal models of various diseases. While tumor immunotherapy may benefit from enhancing costimulatory signaling, control of autoimmune/inflammatory diseases may be achieved by inhibiting these signals [46].

### 3.4. Vaccines

Vaccines make up the largest category of immunotherapy for brain tumors; however, clinical success has been difficult to achieve despite a variety of approaches taken. Vaccines require tumor antigens in the form of whole tumor, tumor lysate, or tumor peptides, in combination with an adjuvant to promote appropriate antigen presentation and priming. While mouse and human studies have shown favorable results, these results have not proven to be effective at generating cure [47,48]. To overcome the potential lack of antigen presentation, dendritic cell (DC) vaccines have been pursued as well [26]. DC isolated from the patient have been pulsed with tumor lysate, tumor peptides, tumor mRNA, or whole tumor cells, and re-administered to patients [26]. DC vaccines account for the majority of ongoing immunotherapy related clinical trials for brain tumors. These clinical trials have been well tolerated by the patients and shown promising signs of immune response correlating with radiographic response and modest improvements in overall survival [49,50,51,52,53]. Due to constraints associated with growing sufficient autologous tumors for therapy, as well as the potential for autoimmune conditions induced by the inclusion of whole tumor cell (containing normal self-proteins) there has been considerable effort to identify glioma-associated antigens (GAAs) and developing synthetic peptides for vaccination based on these antigens. These synthetic peptides can be used in vaccines and administered with adjuvant, as was done with the common mutation EGFRvIII. Clinical studies showed promising signs of efficacy; however, 82% of patients had lost EGFRvIII expression upon recurrence. These data suggest immunoediting is at work and indicate a need to target multiple antigens in vaccines to avoid immune evasion [23]. Multiple GAAs have since been identified and synthetic peptides used to pulse DC for GBM vaccines have demonstrated immunological response and modest effects on clinical outcome [54,55]. The majority of these peptides are MHC I-restricted epitopes generated with the intent to stimulate the CD8 T cell response to tumor [56]. This approach excludes CD4 T cell activation or incorporation of additional immune cell responses.

Multiple trials have shown modest success; however, clinical data only exists for phase I and II trials as we await phase III clinical data. Due to the small number of patients in early phase clinical trials, many of these studies were not scaled to show statistical clinical response. Consequently, many of these studies used biomarkers such as CD8 T cells expansion, ex vivo CTL activity, and delayed-type hypersensitivity (DTH) response to evaluate success of the immunization [49,51,52,54]. Despite multiple papers indicating activation of a Th1 response to vaccine none of these therapies has moved past phase III clinical trials or demonstrated cure in patients. Although the possible reasons for clinical failure of vaccines are numerous, overemphasis on CD8 T cell stimulation may be a factor hindering progress in therapeutic efficacy.

## 4. Evidence for CD8 T Cell-Independent Tumor Clearance in Glioma

Our investigation of a vaccine consisting of an autologous brain tumor lysate mixed with CpG-containing oligonucleotides in an orthotopic mouse model of GBM revealed that while this therapy elicited initial CD8 T cell priming, the response generated was insufficient for tumor clearance [57]. Addition of the costimulatory molecule OX40L fused to the immunoglobulin Fc protein (Fc-OX40L) generated a robust immune response capable of complete tumor clearance in ~70% of animals treated [57]. Importantly, this therapy was able to generate immunological memory to the tumor as demonstrated by complete and rapid tumor rejection upon rechallenge in surviving animals [57]. Further investigation revealed the response generated was dependent on CD4 T cells, B cells, and NK cells [58]. An interesting observation was the massive invasion of granulocytes into the tumor following Fc-OX40L therapy. Further analysis revealed this population was only present in animals receiving Fc-OX40L and did not exhibit suppressive capabilities in ex vivo tests, suggesting they were neutrophils and not myeloid-derived suppressor cells (MDSCs) [58]. The most striking result revealed the mechanism of tumor rejection was independent of CD8 T cells, as depletion of CD8^+^ positive cells or use of CD8 knockout mice did not alter therapeutic outcome [58]. In light of the current dogma surrounding CD8 T cells and cancer immunotherapy these results were surprising and suggest other immune cell populations should be considered when designing new cancer immunotherapies. Another important aspect of our results was a multilayered approach to tumor killing. The involvement of cells from the innate and adaptive immune systems highlights the complexity of a successful immunological response to cancer. Although a CTL response has been considered the benchmark for therapeutic efficacy it is becoming more appreciated that other immune cell populations play equally important roles in tumor models and patients.

The focus on CD8 T cells as the central immune cell for tumor clearance is well reasoned. These cells possess the ability to directly kill tumor cells presenting tumor antigens via MHC I. However, there are a number of limitations to this approach in many cancers. Several cancers, including GBM, downregulate MHC I expression, as evidenced by the absence of MHC I expression on GBM cells that have invaded normal brain tissue [59]. These are the cells that are left after surgical resection and the critical target for immunotherapy. In the absence of MHC I, CD8 T cells are unable to recognize cognate antigen on the target cell and rendered ineffective against tumor cells. Interferon (IFN)-γ can increase MHC I expression on a variety of tumor cells; however, many more barriers must be overcome to generate a successful immune response to GBM [60]. As discussed above the immune status of the CNS has provided significant hurdles to the generation of an effective CTL response. Despite the immune suppression of CD8 T cell response many other immune cells are in the CNS. Additionally, many cell types possess antitumor properties in a variety of tumors.

### 4.1. CD4 T Cells

The helper function of CD4 T cells is necessary to provide crucial cues to surrounding cells and generally orchestrate the immune response to tumors or pathogens [61,62]. The type of response generated by these cells is critical to the efficacy of the response. The emphasis on a Th1 CD4 T cell bias has been considered ideal for generating anti-tumor CD8 T cells. This approach has several limitations, including downregulation of MHC I on the surface of infiltrative brain tumors and the CD8 T cell dampening cytokine milieu of the CNS. Several reports have countered the importance of a Th1 response and provide evidence for diverse mechanisms for tumor rejection independent of CD8 T cells. CD4 T cells themselves have been described as being more efficient at tumor rejection than CD8 T cells [63]. In addition to the indirect mechanism of killing that CD4 T cells are most known for, there is evidence for “cytotoxic” CD4 T cells, particularly in viral infection models [64]. While CD4 T cells have conventionally been identified as CTL promoting Th1 cells or humoral response promoting Th2 cells, the identification of additional subtypes (e.g., Th9, Th17, and regulatory T cells) and ongoing classification of specialized functions of these cells opens the possibility of a more varied and complex response to tumors than previously appreciated. Additionally, CD4 T cells are necessary for the initiation and maintenance of CD8 T cell responses. The absence of CD4 signaling results in progressive loss of CD8 activity over time, leading to a blunted antitumor response [61,62]. Historically, many cancer vaccines and ACT protocols focused on MHC I-restricted epitopes and exclusive transfer of CD8 T cells. Subsequent data has shown the inclusion of tumor reactive CD4 T cells in ACT could enhance persistence of transferred CD8 T cells [65,66]. Additionally, vaccination with the MHC II-restricted epitope for a common glioma mutation in isocitrate dehydrogenase type 1 (IDH1) was successful in a mouse model and CD4 T cell and B cell dependent [67].

Regulatory T cells (Tregs) are a subset of CD4 T cells with the capacity to negatively regulate the immune response, maintaining homeostasis and preventing uncontrolled immune responses and autoimmunity. Accumulation of Tregs is commonly observed in gliomas and an increased proportion of infiltrating Tregs correlates with poor outcome [68]. Tumor-secreting cytokines are responsible for recruitment of naturally occurring Tregs (nTregs) and the conversion of CD4 Th cells into inducible Tregs (iTregs) at the tumor site [69,70]. The suppressive capacity of these cells is a major hurdle to overcome when developing an effective antitumor response. Depletion of Tregs can be accomplished with chemotherapies, such as cyclophosphamide, although not without potential side effects [71]. Inhibition of the suppressor function of Tregs can be achieved through costimulory molecules, such as GITR and OX40 [72,73,74].

### 4.2. Natural Killer (NK) Cells

The role of NK cells in cancer is evident in name and the fact they were identified in 1975 for their unique ability to kill tumors without previous immunization. Since then multiple modalities of NK cell killing of tumor or virally-infected cells have been identified. Unlike other members of the innate immune system the presence of NK cells has been correlated with positive outcomes in cancer patients [75]. NK cells are capable of targeting cells that do not express MHC I or upregulate certain stress molecules. Direct killing of target cells incudes release of perforins and granzymes, binding of TNFR family members, secretion of IFN-γ or nitric oxide [75]. NK cells can also provide a link between the innate and adaptive immune responses by secreting cytokines and chemokines that promote effector cell differentiation [75]. Additionally, NK cells are also able to kill antibody coated target cells through antibody dependent cell-mediated cytotoxicity (ADCC), in which the Fc portion of the immunoglobulin protein binds the Fc receptor (FcR) on an effector cell (including NK cells, macrophages, neutrophils, and DC) [75]. Mouse models continue to provide evidence for the importance of NK cells in tumor clearance and immunotherapy. NK cells can infiltrate the CNS in human GBM and mouse models [73]. Several mouse models have shown the efficacy of NK cell therapy in treating brain tumors, including one study that looked at brain metastasis of melanoma and preferential clearance by NK cells in the context of CD8 knockout animals [76]. Our own studies have demonstrated the requirements of NK cells for effective immunotherapy [58]. Our results indicate the potential role of ADCC in a murine model of GBM, as efficacy is reduced in animals deficient for FcR [58]. These findings provide evidence for the potential of NK cell contributions to effective immunotherapy regimens.

### 4.3. B Cells

The majority of immunotherapeutic approaches have attempted to activate the adaptive arm of the immune system with most of the attention given to the role of T cells, while B cells have largely been ignored. The advantage to utilizing the adaptive immune response includes the generation of memory and antigen specificity. B cells that encounter antigen can differentiate into antibody-secreting plasma cells or memory B cell, which confer protection upon secondary encounter with antigen [77]. ADCC is a potential mechanism of tumor destruction by the immune system and appropriate activation and differentiation of B cells is necessary for this to occur. Although the role of B cells as APCs is less studied than DC, B cells can also present antigen to T cells, contributing to T cell activation [77]. Passive diffusion of large proteins, including immunoglobulins, into the brain is restricted by the BBB and thus the role of an antibody response in the efficacy of immunotherapy for brain tumors has been diminished [78]. Paradoxically, there is a correlation between the presence of tumor-reactive antibodies in GBM patients and survival [79]. This observation is supported by mouse models of GBM suggesting B cells are crucial for efficacy of immunotherapy [58,80].

### 4.4. Macrophages

Much of the attention given to innate immune cells in response to tumors has focused on the tumor promoting or immune suppressive capacity of these cells. Macrophages can be divided into two main subsets, conventionally activated M1 macrophages or alternatively activated M2 macrophages [81]. M1 macrophages promote a pro-inflammatory pathway, conversely M2 or tumor-associated macrophages (TAM) can infiltrate the tumor site and contribute to the immune suppressive microenvironment and are negatively associated with patient outcome [81,82]. Several publications have raised the possibility of utilizing macrophages in the antitumor immune response. Administration of an agonist anti-CD40 antibody to mice and human patients with pancreatic cancer demonstrated robust antitumor response [83]. The anti-CD40 antibody was expected to promote antigen presentation by DC leading to enhanced CTL activity. However, the study showed an influx of tumoricidal macrophages and tumor regression independent of CD4 of CD8 T cells [83]. Microglia in the brain have similar immune suppressive properties in brain tumors and compose a large proportion of most brain tumors [82,84]. Many therapeutic approaches have focused on eliminating these TAMs. An alternative method to elimination may be to re-direct these cell populations to promote tumor elimination. CSF-1R inhibition can eliminate M2 macrophages in animal models; however, administration of a CSF-1R inhibitor in a mouse model of GBM did not eliminate TAMs. Instead the CSF-1R inhibitor changed the functionality of these cells, abolishing the immune suppressive properties and promoting an anti-tumor response [85]. Additional studies have shown antitumor activity of macrophages through various mechanisms including nitric oxide (NO), ADCC, or TNF-related apoptosis inducing ligand (TRAIL) induced death [86,87,88].

### 4.5. Neutrophils

Although little is known about the role of neutrophils in the context of cancer, tumor-associated neutrophils (TAN) have correlated with poor prognosis in many cancer patients, including glioma, and have been viewed as a potential target in immunotherapeutic approaches [89,90,91]. However, much like macrophages, the functionality of these cells depends of the microenvironment, with polarization of these cells into pro-tumor (N2) or anti-tumor (N1) neutrophils dependent of the cytokine milieu [92]. The variable role of neutrophils in cancer biology has been reviewed elsewhere [92] but like M2 macrophages and myeloid-derived suppressor cells (MSDC; discussed below), N2 neutrophils appear to possess the potential for an anti-tumor response if given the correct cues and neutrophils have shown direct tumoricidal activity through production of reactive oxygen species, ADCC, and FasL-induced apoptosis [92,93]. Our previous observation correlating neutrophil infiltration of tumors with increased survival [58] raises the possibility of therapeutically re-educating distinct cell populations to promote the antitumor response as was seen for macrophages and MDSC [85,94,95,96].

### 4.6. Myeloid-Derived Suppressor Cells (MDSC)

MDSC constitute a heterogeneous population of immature myeloid cells in which terminal differentiation (into DC, macrophages, or neutrophils) is blocked by tumor-derived cytokines [97]. Consequently, elevated levels of circulating or tumor-localized MDSC have been associated with poor prognosis in a number of tumors [98]. Immune suppressor cell types are largely attributed to the failure of many immunotherapeutic approaches and strategies to ablate these cells are viewed as the path to more effective immunotherapies. Chemotherapies, such as 5-fluorouracil, can reduce the number of circulating MDSC, but are not without significant side effects [81]. Conversely, signals promoting maturation reduce the immunosuppressive function of MDSC and promote antitumor immunity [94,95,96]. Due to the phenotypic similarities between MDSC and neutrophils the two populations are difficult to distinguish without utilizing ex vivo suppression assays. As a result populations resembling MDSC have been viewed as deleterious and most efforts have focused on elimination rather than redirecting their functionality.

### 4.7. Mast Cells

An interesting study using IL-9 therapy in a mouse model of melanoma was able significantly inhibit tumor growth. Intriguingly, this result was independent of T cells, but dependent on mast cells [99]. Similarly, TLR2 stimulation of mast cells also demonstrated antitumor activity in a murine melanoma model, in which mast cells were able to recruit NK and T cells and production of IL-6 had direct antitumor effects [100]. Like the other innate cells reviewed here mast cell involvement in cancer has presented conflicting reports of both pro- and anti-tumor roles. Anti-tumor properties of mast cells, such as killing tumors directly through ADCC or release of TNF and indirectly by chemokine recruitment or cytokine-mediated modulation of effector cells, have been reported [100,101]. The involvement of mast cells in tumor biology and immunotherapy has been more extensively reviewed elsewhere [101]. There is a shortage of data concerning mast cells in gliomas; however, one publication showed the presence of mast cells in a mouse model of glioma as well as human patients [102]. A greater number of mast cells were seen in human GBMs compared to grade II gliomas, suggesting a tumor-promoting role of mast cells [102]. The role of mast cells in gliomas and the potential to therapeutically alter their function to promote tumor clearance remains to be seen.

## 5. Conclusions: Rethinking the Strategy

**Figure 1:**
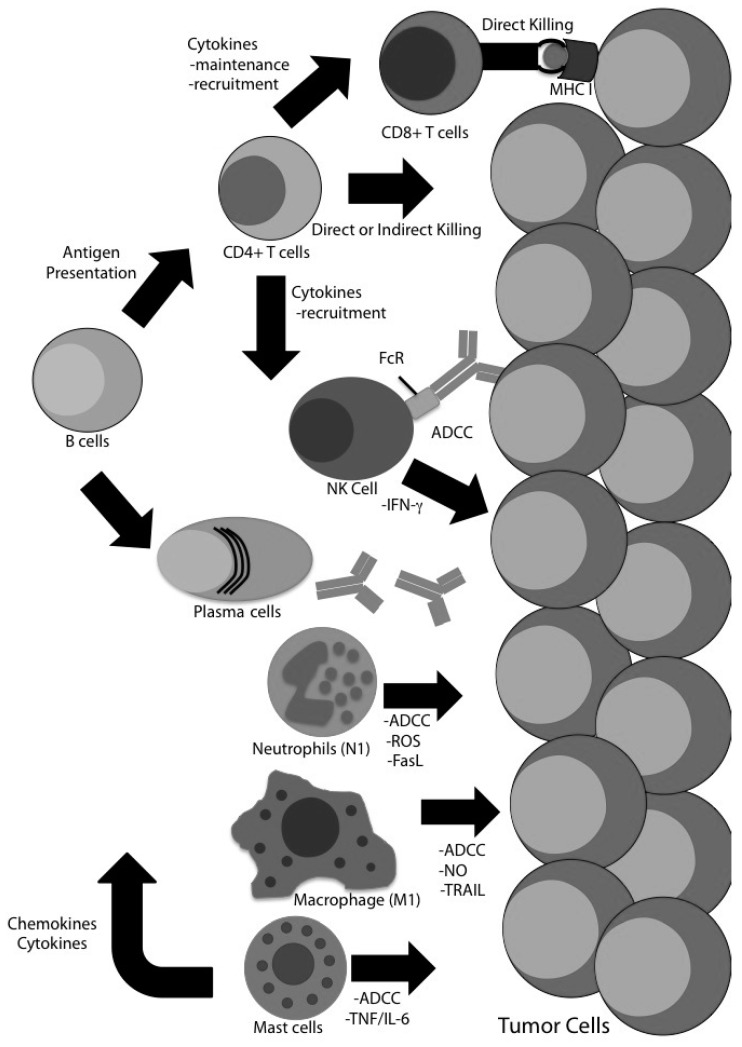
Recruitment of multiple immune cell types for enhanced tumor destruction. While the majority of immunotherapies focus on CD8 effector cells, a robust antitumor immune response involving a plethora of appropriately polarized and activated immune cells could potentiate tumor clearance. Multiple members of the immune system have diverse mechanisms for tumor clearance. Recruitment of a combination of immune responses may generate an overwhelming antitumor immune response, overcoming immune suppression and resulting in tumor clearance.

The compiled data suggests the conventional approach to immunotherapy by stimulating a cytolytic CD8 T cell response may be overly restrictive, especially for tumors of the CNS. CD4 T cells orchestrate the immune response by secreting cytokines necessary for recruitment, survival, and polarization. Without the appropriate CD4-derived signals CD8 T cell responses diminish over time. With evidence that innate immune cells (under the right conditions) can be appropriately directed to kill tumor cells, therapies that include these cells should be pursued. Tumors utilize a multifactorial approach to immune evasion—including (but not limited to) the lack of antigen presentation and costimulation, secretion of immune suppressive cytokines, and recruitment of immune suppressive cells (MDSC, TAM, TAN, Tregs). Therefore, immunotherapy should offer an equally broad response. By broadening the approach, perhaps we will be able to overwhelm the tumor and tip the balance in favor of the immune system (Figure 1). Therapies incorporating multiple pathways present exciting opportunities to stimulate multiple cell types. Adjuvant therapies, such as CpG, are used to stimulate DC for efficient priming of T cells, but they can also mature MDSC. Costimulatory molecules, such as OX40L or GITRL, have dual roles in immune modulation. In addition to stimulating effector T cells, ligation of costimulatory receptors inhibit Treg function and break tolerance [72,74,103]. These observations present exciting potential for the use of combination therapies to generate a comprehensive immune response.

Our results demonstrating a CD8 T cell-independent mechanism of tumor clearance in a mouse model of GBM does not suggest CD8 T cells are not necessary for tumors outside the CNS or that CD8 T cells cannot be activated in response to CNS tumors. Instead, when considered along with the previous characterization of the CNS immunology and earlier research suggesting alternative means of tumor eradication, these results should promote the incorporation of additional immune targets into immunotherapy and contribute to the elucidation of effective antitumor immune response for glioma. The failure of current therapy for GBM may be due to a lack of incorporation of the full immune response. While the unique microenvironment of the brain could elevate the importance of this strategy it remains to be seen if alternative mechanisms of antitumor response are tumor- or location-specific. Therapeutic approaches should take into account the state of the tumor microenvironment and the immunological bias of the organ in which they reside.
